# Post-stroke Fatigue and Depressive Symptoms Are Differentially Related to Mobility and Cognitive Performance

**DOI:** 10.3389/fnagi.2017.00343

**Published:** 2017-10-31

**Authors:** Bradley J. MacIntosh, Jodi D. Edwards, Mani Kang, Hugo Cogo-Moreira, Joyce L. Chen, George Mochizuki, Nathan Herrmann, Walter Swardfager

**Affiliations:** ^1^Sunnybrook Research Institute, Toronto, ON, Canada; ^2^University of Toronto, Toronto, ON, Canada; ^3^Canadian Partnership for Stroke Recovery, Toronto, ON, Canada; ^4^Ottawa Hospital Research Institute, Ottawa, ON, Canada; ^5^Federal University of São Paulo, São Paulo, Brazil; ^6^University Health Network Toronto Rehabilitation Institute, Toronto, ON, Canada

**Keywords:** stroke, fatigue, depression, mobility, cognition, mediation, factor analysis

## Abstract

**Background:** Fatigue and depressive symptoms are common and often inter-related stroke sequelae. This study investigates how they are related, directly or indirectly, to mobility and cognitive outcomes within 6 months of stroke.

**Methods:** Participants were recruited from 4 stroke centers in Ontario, Canada. Post-stroke fatigue was assessed using the Fatigue Assessment Scale (FAS). Depressive symptoms were screened using the Center for Epidemiological Studies Scale for Depression (CES-D). Factor analyses were used to construct scores from mobility (distance traveled during a 2-min walk test, Chedoke-McMaster Stroke Assessment leg score, and Berg Balance Scale total score) and cognitive (Montreal Cognitive Assessment, Trail-Making Tests A and B, and five-word free recall) tests. Direct associations were assessed in linear regression models and indirect effects were assessed in path models. Covariates were age, sex, education, antidepressant use, days since stroke, and stroke severity (National Institute of Health Stroke Severity Scale score).

**Results:** Fatigue and depressive symptoms were highly correlated (*r* > 0.51, *p* < 0.0001). Depressive symptoms were associated with cognition (β = −0.184, *p* = 0.04) and indirectly with mobility, mediated by fatigue (indirect effect = −0.0142, 95% CI: −0.0277 to −0.0033). Fatigue was associated with mobility (β = −0.253, *p* = 0.01), and indirectly with cognition, mediated by depressive symptoms (indirect effect = −0.0113, 95% CI: −0.0242 to −0.0023).

**Conclusions:** Fatigue and depressive symptoms are related distinctly to cognitive and mobility impairments post-stroke. Fatigue was associated with poorer lower limb motor function, and with cognition indirectly via depressive symptoms.

## Introduction

Fatigue and depressive symptoms are common, complex, and incompletely understood stroke sequelae (Wu et al., 2014; Duncan et al., 2015; Ponchel et al., 2015). Fatigue is characterized by persistent or extreme tiredness, weakness or exhaustion, aversion to effort, and difficulty in initiating or sustaining voluntary activities (Chaudhuri and Behan, 2004). Some of these symptoms can overlap with post-stroke depression, posing difficulty in disentangling their impacts on recovery. Moreover, the syndromes often co-occur, with one third of stroke survivors experiencing both fatigue and depression (Choi-Kwon and Kim, 2011; Tang et al., 2013, 2014; Wu et al., 2014). Co-occurrence might be expected, since post-stroke fatigue and post-stroke depression share common risk factors, notably stroke severity and functional impairments (Andersen et al., 1995; Ponchel et al., 2015). Additional clinical risk factors for both include recurrent stroke, premorbid psychiatric illness, social distress, isolation, and sometimes sex (Andersen et al., 1995; Ponchel et al., 2015).

Fatigue and depressive symptoms are sufficiently prevalent to pose tangible barriers to recovery and rehabilitation. Post-stroke depression increases the risk of cognitive impairment (Kauhanen et al., 1999), and it appears to contribute to mobility outcomes (van de Port et al., 2006). Fatigue influences mobility (Ponchel et al., 2015), as measured using the Berg Balance Scale (BBS) or mobility tests (Michael et al., 2006; Duncan et al., 2015). There remains a need to dissect the complex inter-relationships between these prevalent sequelae and cognitive and mobility outcomes (Mandliya et al., 2016). There is little evidence that post-stroke fatigue is directly associated with cognition; however, a recent synthesis of evidence suggested that fatigue may heighten the influence of depressive symptoms (Ponchel et al., 2015), and therefore, there may be an indirect relationship between fatigue and cognition that is mediated by depressive symptoms. It is of clinical importance to identify primary treatment foci because pharmacological treatments for post-stroke depression, fatigue, and cognitive impairment may have unexpected, complementary or antagonistic effects on functioning in other domains of recovery.

This study assesses direct and indirect effects of fatigue and depressive symptoms on mobility and cognitive outcomes within 6 months of stroke. These outcomes were considered because they are important components of stroke recovery, and they might be viewed as largely distinct disabilities. We hypothesized that depressive symptoms would be related to cognition independently of fatigue, and that an indirect effect of depressive symptoms on mobility would be mediated by fatigue. We also hypothesized that fatigue would be related to mobility independently of depressive symptoms, and that an indirect effect of fatigue on cognition would be mediated by depressive symptoms.

## Methods

### Participants

Participants were part of the Rehabilitation Affiliates Study, a multi-site investigation on physical and cognitive recovery after stroke in Toronto (Middleton et al., 2014). We report cross-sectional data from participants who presented to their first visit within 180 days of acute stroke. Inclusion criteria: primary diagnosis of ischemic or haemorrhagic stroke, ability to speak and understand English, completed assessments for depression, cognitive status, and stroke severity. Participants with incomplete cognitive or mobility data were excluded from those analyses. All subjects gave written informed consent in accordance with the Declaration of Helsinki. The protocol was approved by the Research Ethics Board of Sunnybrook Health Sciences Centre.

### Screening for depressive symptoms and fatigue

The Center for Epidemiological Studies Depression scale (CES-D) is a 60-point self-report instrument that assesses the presence and severity of depressive symptoms over the past week, used extensively in stroke (Bensimon et al., 2014; Swardfager et al., 2014). The Fatigue Assessment Scale (FAS) is also a self-report instrument used in stroke research (Duncan et al., 2015), with 10 items to score aspects of fatigue, each rated from 1 to 5. A higher FAS score indicates greater fatigue.

### Mobility assessments

Mobility was assessed using 3 measures: BBS score (Berg et al., 1992), the Chedoke-McMaster Stroke Assessment leg (CMSA-leg) score (Gowland et al., 1993), and the distance traveled during a 2 min walk test (**Figures 2A,C**). Exploratory factor analysis was used to produce a normally distributed composite mobility score based on lower limb function, combining variance common to each mobility test to minimize the impact of measurement errors inherent to each test, to account for ceiling effects on the Berg, for floor and ordinal effects in the CMSA-leg, and to produce a single mobility outcome score.

### Cognitive assessments

A composite score was produced using exploratory factor analysis of four cognitive measures (**Figures 2B,D**) to capture cognitive status, attention and psychomotor processing speed, executive function and verbal memory: the Montreal Cognitive Assessment (MoCA), a validated screen for cognitive impairment after stroke (Cumming et al., 2013), the times to completion of the Trail Making Test parts A (Trails-A) and B (Trails-B) and the number of words correctly recalled during a 5 Word Delayed Free Recall task.

### Statistics

Composites were generated using maximum likelihood exploratory factor analysis (“factanal” in R version 3.1.2). Outcomes were derived from all participants who had 3 mobility or 4 cognitive measures. The factor loadings used the Thomson's regression method.

Linear regression models were run for the two composites separately. CES-D and FAS were independent variables. Covariates included a priori were age, National Institute of Health Stroke Severity (NIHSS) scale, days post-stroke and use of antidepressant medications. Years of education was covaried in the cognitive model. Path analysis assesses whether one independent variable influences a dependent variable indirectly via a second mediating variable. Direct and indirect influences of CES-D and FAS on the composite outcome measures were assessed using the PROCESS macro (Hayes, 2013) for SPSS. Ten thousand bootstraps produced 95% confidence intervals (CI) on the path coefficient parameter estimates (**Figure 4**). Mediated (indirect) effects were considered significant if the 95% CI for their parameter estimates did not cross zero.

## Results

**Figure 1:**
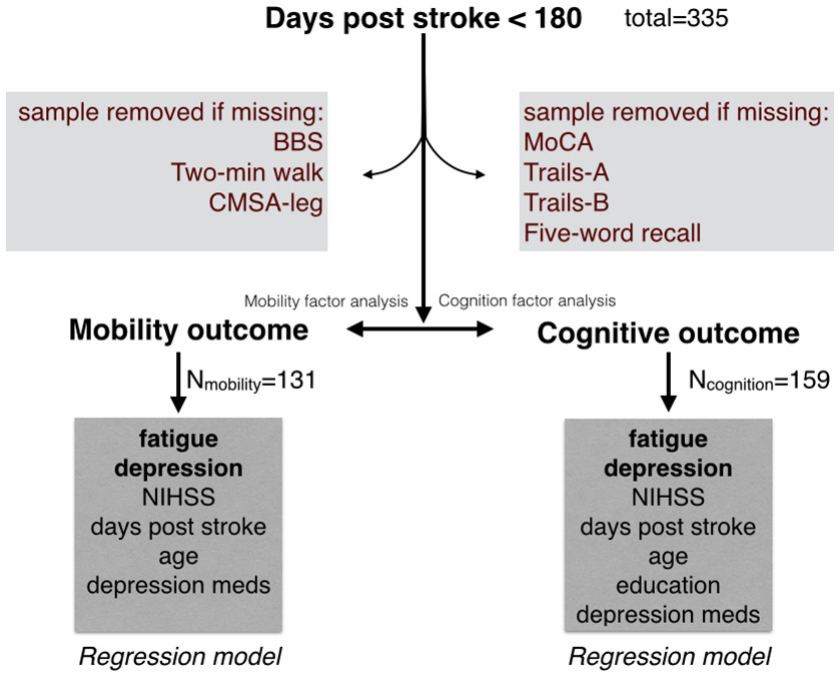
Initial sample from the Rehab Affiliates study, and the sub-groups that were formed to test hypotheses. Only complete records were considered in the regression models. Factor analysis was performed to generate a single composite score for mobility, and similarly a single composite score for cognition.

From 335 Rehab Affiliates participants, 131 had complete mobility data and 159 participants had complete cognitive data (Figure 1). The characteristics of the entire cohort and both subgroups are shown in Table 1. FAS and CES-D scores were significantly correlated (Mobility subgroup: *t*_(1, 129)_ = 8.6, *p* < 0.001; Cognitive subgroup: *t*_(1, 157)_ = 7.5, *p* < 0.001).

**Table 1:** Participants details.

**Description**	**FULL Sample**	**MOB sub-group**	**COG sub-group**	**FULL vs. MOB**	**FULL vs. COG**	**MOB vs. COG**
Sample, number	335	131	159	n.a.	n.a.	n.a.
Males, number	195	78	109	n.s.	n.s.	n.s.
Age, median	68	65	64	n.s.	n.s.	n.s.
NIHSS, median	4	3	4	n.s.	n.s.	0.04
Days post stroke, median	37	34	34	n.s.	n.s.	n.s.
Type of stroke, %						
Ischemic	66.3	66.2	63.3		n.s.	
Lacune	7.7	8.1	9.5			
Hemorrhagic	18.1	16.2	18.9			
TIA	5.1	6.6	6.5			
Affected side of body, %						
Right	36.0	41.2	38.5		n.s.	
Left	56.4	49.3	55.6			
Both	5.7	8.8	5.3			
Neither	1.4	0.0	0.0			
Hypertension history, %	79.6	81.6	81.7	n.s.	n.s.	n.s.
Hypertension medication, %	58.1	55.1	64.5	n.s.	n.s.	n.s.
Body Mass Index, median	25.3	25.3	25.0	n.s.	n.s.	n.s.

There were no significant differences between the mobility and the full sample in demographics, medications or other characteristics assessed (Table 1). FAS was correlated with use of antidepressant medications (*r* = 0.18, *p* = 0.036) but not NIHSS (*r* = 0.15, *p* = 0.10), nor age (*r* = −0.024, *p* = 0.78). CES-D was not correlated with NIHSS (*r* = −0.04, *p* = 0.66), nor age (*r* = −0.09, *p* = 0.32). There were no significant differences between the cognitive sample and the whole sample, or between the cognitive and mobility samples in demographics, medications or other characteristics assessed, except NIHSS scores (Table 1).

### Effects of fatigue and depressive symptoms on the mobility composite score

**Figure 2:**
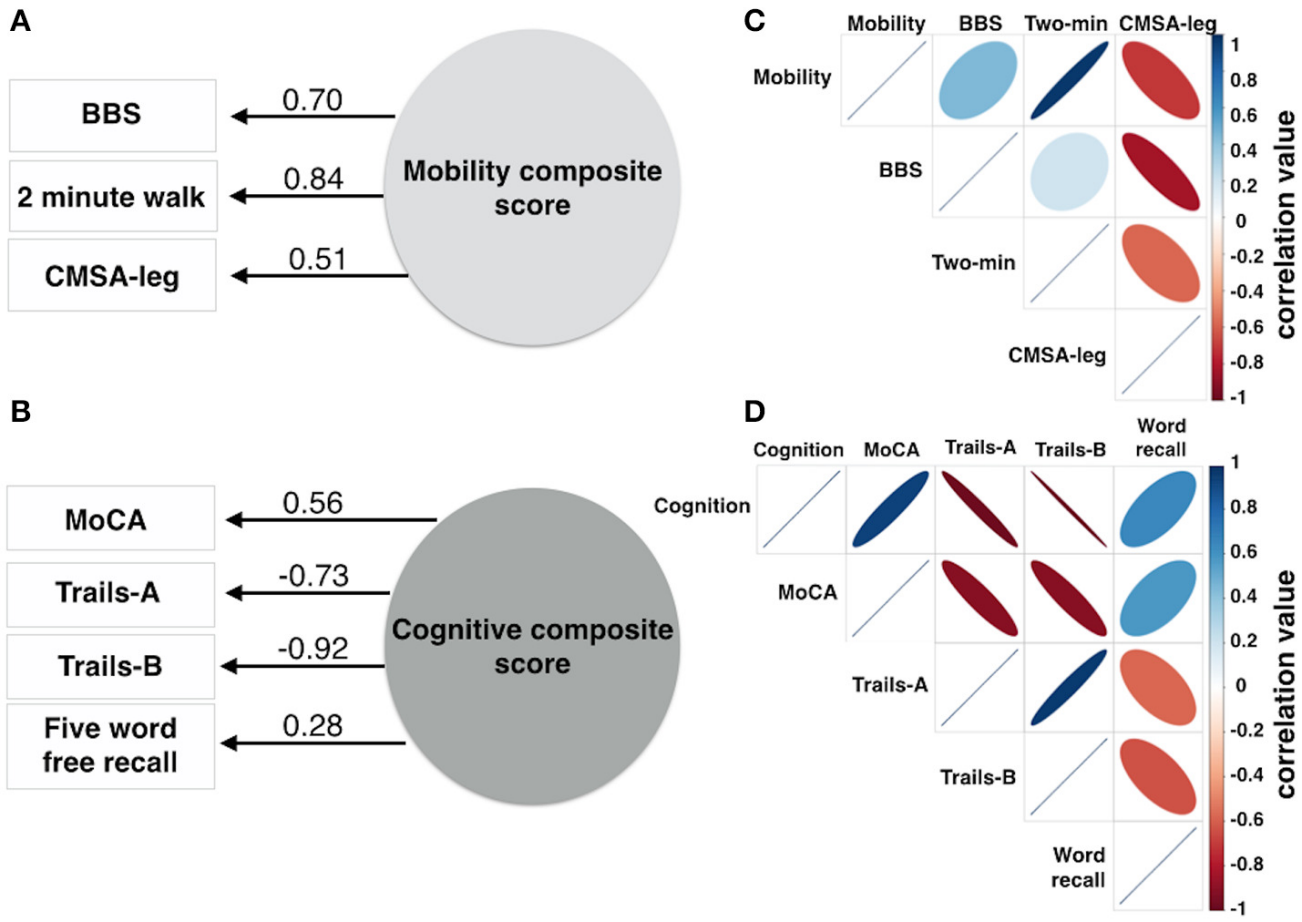
Composite scores for mobility and cognition were derived from 3 or 4 variables, respectively. Factor analysis produced one factor for mobility **(A)** and one factor for cognition **(B)**. The numbers adjacent to the lines in **(A,B)** denote the factor loadings onto the composite score. The correlation matrix describes the individual correlation for the mobility data **(C)** and cognitive data **(D)**. The color bars in **(C,D)** represent correlation coefficient values ranging from −1 to 1. The size of the ellipse indicates the degree of shared variance between each of the variables (wide ellipse, low correlation; narrow ellipse, high correlation). BBS, Berg Balance Scale; CMSA-leg, Chedoke-McMaster Stroke Assessment leg score.

**Figure 3:**
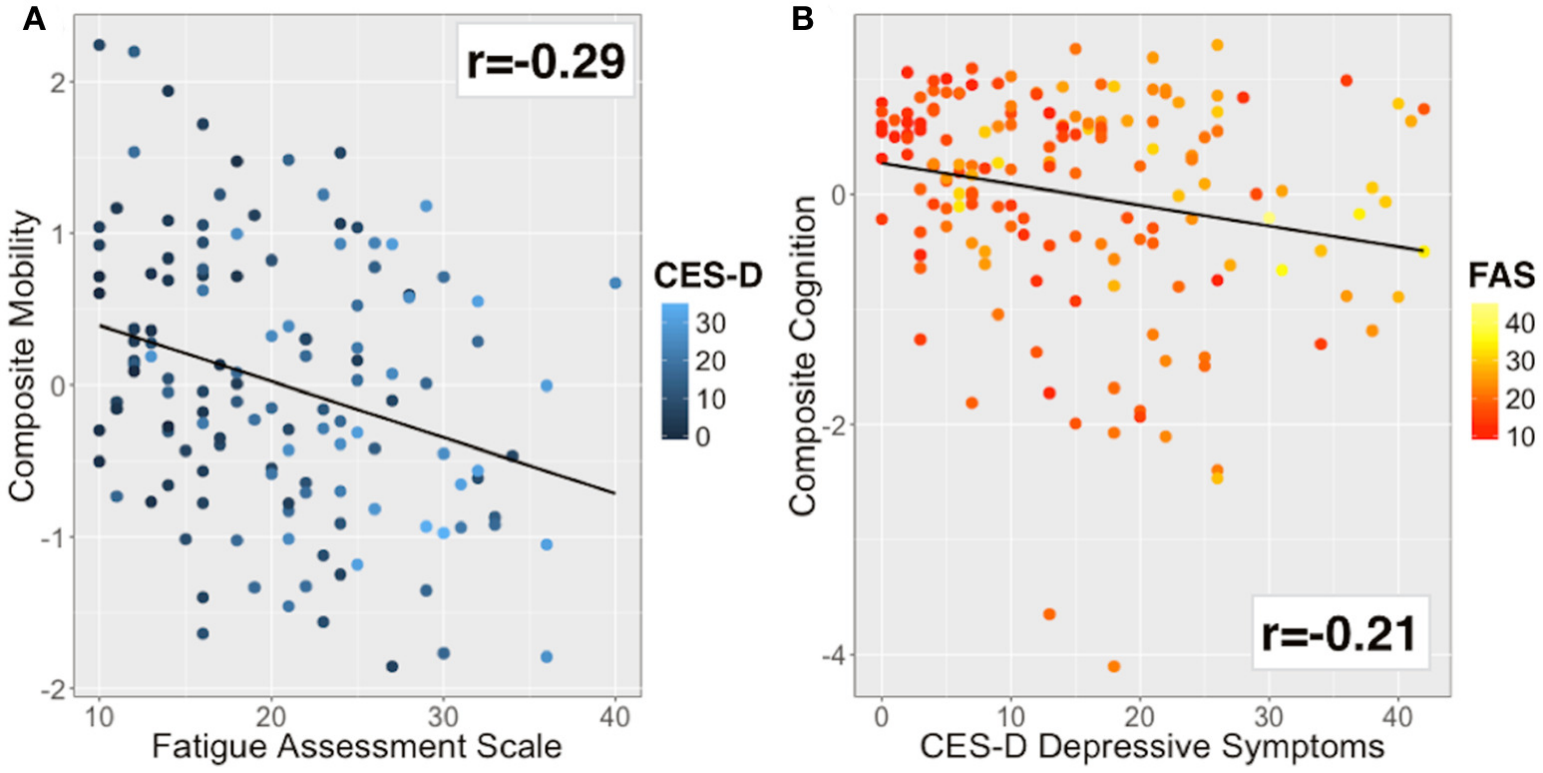
Bivariate correlations for the main outcome and explanatory variables. **(A)** Mobility was inversely correlated with fatigue (*r* = −0.29). Color of data points based on CES-D (blue to cyan scale). **(B)** Cognitive performance was inversely correlated with depressive symptoms (*r* = −0.21). Color of data points based on FAS (red to yellow scale).

**Figure 4:**
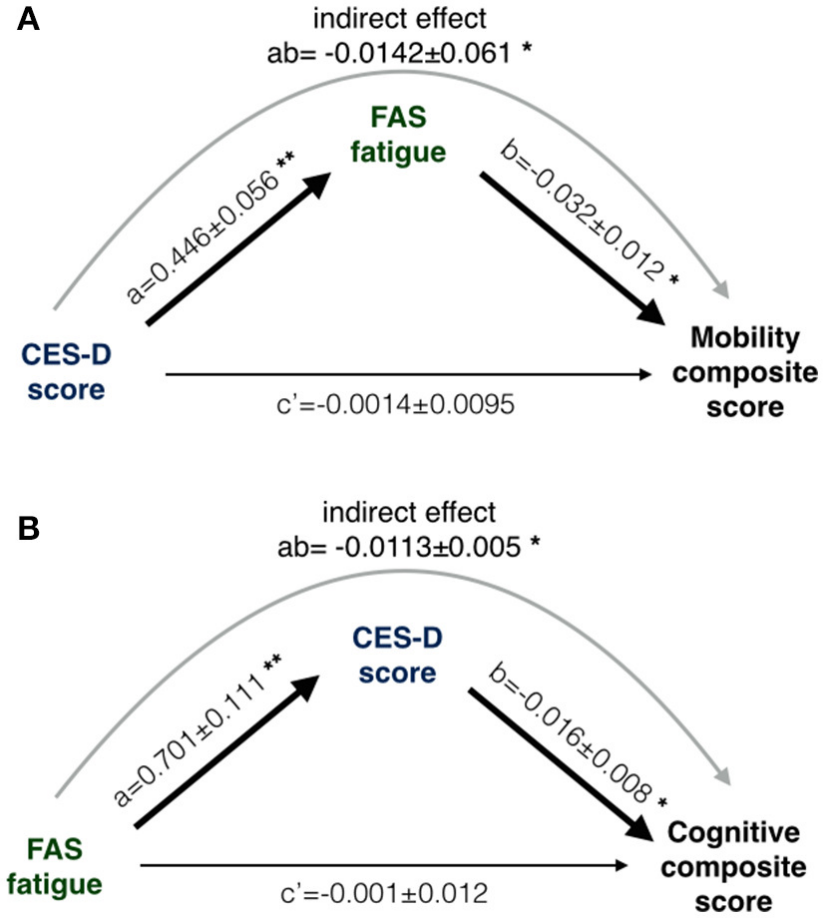
The results of two mediation path analysis models. One path model **(A)** was designed to explain between-subjects differences in mobility; the other path model **(B)** attempted to explain between-subjects cognitive differences. Coefficients in the path model are listed as a, b and c', where a and b are part of the indirect path and c' is the direct path adjusted for the indirect path. **(A)** CES-D was not directly associated with mobility (c' non-significant) but the indirect path was significant (denoted as ab). **(B)** FAS was not directly associated with cognition (c' non-significant) but the indirect path was significant (denoted as ab). Statistical criteria for significance are ^*^p < 0.05 and ^**^p < 0.01.

The mobility composite explained 49% of the mobility variance with loadings of 0.70, 0.84, and 0.51 for BBS, Two Minute walk and CMSA-leg, respectively (Figure 2A). Mobility and FAS were associated independently (Table 2A, β = −0.253, *p* = 0.01; see Figure 3A). The model, including all predictors, explained 21.5% of the variance in mobility. Mediation analysis (Figure 4A) revealed no significant direct effect of CES-D on mobility (coefficient = −0.0014, 95% CI: −0.0201 to 0.0174); however, there was significant indirect effect of CES-D on mobility that was mediated by FAS scores (coefficient = −0.0142, 95% bootstrap CI: −0.0277 to −0.0033).

**Table 2:** Model results for **(A)** mobility and **(B)** cognitive outcomes.

**Model**	**Coefficient**	**SE**	**β**	***P***
**A. PREDICTORS OF THE MOBILITY COMPOSITE SCORE**				
FAS	−0.032	0.012	−0.253	0.01^*^
CES-D	−0.001	0.010	−0.014	0.89
Age	−0.020	0.005	−0.294	< 0.001^*^
NIHSS	−0.109	0.031	−0.278	< 0.001^*^
Days post-stroke	−0.003	0.002	−0.087	0.27
Anti-Depressant	−0.024	0.160	−0.012	0.88
**B. PREDICTORS OF THE COGNITIVE COMPOSITE SCORE**				
CES-D	−0.016	0.008	−0.184	0.04^*^
FAS	0.001	0.012	0.007	0.93
Age	−0.022	0.005	−0.321	<0.001^*^
NIHSS	−0.088	0.024	−0.283	< 0.001^*^
Days post-stroke	−0.004	0.021	−0.154	0.04^*^
Years Education	0.128	0.020	0.048	0.52
Anti-Depressant	0.146	0.155	0.071	0.35

***A**: Overall model R^2^ = 0.251, adjusted R^2^ = 0.215, F_(6, 124)_ = 6.93, p < 0.001*.

***B**: Overall model R^2^ = 0.242, adjusted R^2^ = 0.207, F_(7, 151)_ = 6.90, p < 0.001*.

* *p < 0.05 statistically significant. Standard error (SE)*.

### Effects of depressive symptoms and fatigue on the cognitive composite score

The cognitive composite score explained 42% of the cognitive variance with loadings of 0.56, −0.73, −0.92, and 0.28 for MoCA, Trails-A, Trails-B and Five Word Free Recall, respectively (Figure 2B). Cognition and CES-D scores were independently associated (Table 2B, β = −0.184, *p* = 0.04; see Figure 3B). The model, including all predictors, explained 20.7% of the variance in cognition. Mediation analysis (Figure 4B) revealed no significant direct effect of FAS on cognition (coefficient = 0.0010, CI: −0.0232 to 0.0252). There was a significant indirect effect of FAS on cognition that was mediated by CES-D (coefficient = −0.0113, 95% CI: −0.0242 to −0.0023).

## Discussion

Post-stroke fatigue was associated with lower limb mobility, while post-stroke depressive symptoms were associated with cognitive performance. In mediation analyses, fatigue was associated with poorer cognitive performance only insofar as it was related to depressive symptoms. Similarly, depressive symptoms were associated with poorer mobility performance only insofar as participants experienced symptoms of fatigue. Previously, depression has been suggested to contribute to a decline in mobility in the first few years post-stroke (van de Port et al., 2006). Fatigue might be assessed as a mediator of this longitudinally. Post-stroke fatigue and depression had overlapping yet distinct effects on functional outcome measures; these findings add to a path analysis study on disability among stroke survivors (Mandliya et al., 2016). The current results underscore the importance of recognizing fatigue clinically, and the need to understand the underlying pathophysiology. Its importance is also highlighted by a high prevalence and persistence, remaining elevated in one study at 6 year follow-up (Elf et al., 2016).

The etiologies of fatigue post-stroke may be heterogeneous, associated with sleep disturbances, symptoms of depression and anxiety, poor coping, loss of control, and with other emotional and behavioral disturbances (Barritt and Smithard, 2011; Wu et al., 2014). Sleep disturbances are particularly notable because obstructive sleep apnea (OSA) is highly prevalent in stroke, it predicts poorer recovery, and it is not consistently identified (Swartz et al., 2016). While here we show that fatigue rooted in depressive symptoms can impact motor recovery, identifying additional comorbidities that contribute to fatigue may identify salient clinical foci for treatment that impact mobility outcomes (Sandu et al., 2015). This is particularly important since targeting post-stroke fatigue symptoms directly has been met with limited effectiveness (Wu et al., 2015).

Post-stroke depression has been associated with stroke type, stroke severity and recurrent stroke, but typically not age nor gender (Jiao et al., 2016; Wei et al., 2016). Some evidence suggests that serotonin transporter and brain-derived neurotrophic factor (BDNF) deficiencies may confer susceptibility (Robinson and Jorge, 2016). Roles of these systems in neurogenesis and neuronal survival in brain regions that subserve motor learning, memory and executive function might link mood and cognitive changes. Lesions that interrupt projections from the midbrain and brainstem (e.g., raphe nucleus, locus coeruleus, substantia nigra, nucleus basalis of Meynert), seeding the cortex with serotonin, norepinephrine, dopamine, and acetylcholine, might also produce both depressive and cognitive symptoms (Loubinoux et al., 2012). Some evidence suggests that inflammation might also contribute to post-stroke cognitive impairment (Rothenburg et al., 2010; Gold et al., 2011; Di Napoli et al., 2012; Slevin et al., 2015). An inflammatory hypothesis for post-stroke depression yielded both positive (Yang et al., 2010; Jiao et al., 2016) and negative (Noonan et al., 2013; Bensimon et al., 2014) results, as reviewed previously (Becker, 2016); recent studies suggest a more robust relationship between inflammation and fatigue (Bensimon et al., 2014), which might be useful to direct future intervention trials (Barritt and Smithard, 2011). For instance, although fluoxetine treatment alleviated post-stroke depression, it did not improve fatigue (Choi-Kwon et al., 2007), whereas treatment for, or successful resolution of, depressive symptoms tend to improve cognition (Doraiswamy et al., 2003).

Fatigue ratings represent manifestations of muscle (peripheral) fatigue and/or central fatigue, which may have different relationships with clinical, biological and behavioral characteristics. Lower physical activity, or reduced output from motor centers affected by stroke, might lead to muscle deconditioning (Marzolini et al., 2016). Post-stroke fatigue does not appear to be related to white matter lesions, atrophy nor stroke type, and lesion location findings have been inconclusive (Kutlubaev et al., 2012; Ponchel et al., 2015); however, post-stroke fatigue may be associated with lesions to the cerebellum, corona radiata, basal ganglia and internal capsule (Kutlubaev et al., 2012; Wei et al., 2016). Although findings have been mixed, meta-analyses suggest a higher likelihood of post-stroke depression with lesions to the right hemisphere (Wei et al., 2015), left basal ganglia or frontal areas (Robinson and Jorge, 2016). Differentiating the underlying pathobiology of fatigue and depression will be important to inform personalized treatments that address particular barriers to cognitive and mobility recovery.

This study generated two holistic stroke recovery outcome measures based on highly sensitive and ecologically valid tests. The approach may discard portions of variance unique to the individual indicators while reinforcing features common to multiple related variables. Capturing the common variance increases the signal-to-noise ratio, while also reducing outlier, ceiling, floor and other spurious effects, as well as reducing the number of multiple comparisons. The approach may prove particularly useful in stroke recovery research where patient heterogeneity is common; for instance, a specific impairment related to stroke may produce a poor result on a single test but not another test intended to measure the same underlying construct.

As potential limitations, we excluded participants with missing data, which reduced the sample size for the mobility and cognitive subgroups, which may have introduced selection biases. Also, the study was cross-sectional; the path analysis results cannot be used to infer causal relationships. Patients were selected within 6 months of stroke, and longer-term studies will also be required. Neuroimaging and fluid biomarker data were unavailable; therefore we are unable to address possible neuroanatomical and inflammatory bases for the current findings (Macintosh and Graham, 2013; Bensimon et al., 2014). Although the FAS is a well-established measure, we cannot comment on how the findings might map onto other fatigue instruments. Similarly, the CES-D cannot replace a clinical diagnosis of a depressive episode; however, it has been used extensively in adults living with stroke (Swardfager et al., 2014). Finally, the BBS probes functional mobility, analogous to the Rivermead Mobility Index in stroke, but it suffers from a ceiling effect in low impairment individuals (Blum and Korner-Bitensky, 2008); we mitigated this effect by hypothesis testing on a composite mobility score.

In summary, this study used inferential path modeling to dissect distinct outcomes related to fatigue and depressive symptoms within 6 months of stroke. Though often related, these stroke sequelae exerted differential influences on cognitive and mobility recovery measures. The results implicate additional screening for barriers to recovery in rehabilitation, since depression may contribute to cognitive recovery while comorbidities that lead to fatigue may portend poorer recovery of motor performance.

## Author contributions

BM, HC, and WS designed the study. BM, JE, HC, and WS conducted analyses. BM, JE, JC, GM, NH, and WS interpreted the findings. WS, JE, MK, and BM drafted the manuscript. All authors reviewed, contributed to and approved the manuscript.

### Conflict of interest statement

The authors declare that the research was conducted in the absence of any commercial or financial relationships that could be construed as a potential conflict of interest.
